# Giant right atrial Myxoma associated with acute coronary syndrome

**DOI:** 10.1093/omcr/omaf193

**Published:** 2026-01-28

**Authors:** Hind Hibatouallah, Zineb Mehssani, Rochde Sayah, Rokya Fellat, Nadia Fellat

**Affiliations:** Cardiology A Department, Ibn Sina University Hospital Center, Mohammed V University of Rabat, Lamfadel Cherkaoui Street, 10005, Rabat, Morocco; Cardiology A Department, Ibn Sina University Hospital Center, Mohammed V University of Rabat, Lamfadel Cherkaoui Street, 10005, Rabat, Morocco; Cardiovascular Surgery Department B, Ibn Sina University Hospital Center, Mohammed V University of Rabat, Lamfadel Cherkaoui Street, 10005, Rabat, Morocco; Cardiology A Department, Ibn Sina University Hospital Center, Mohammed V University of Rabat, Lamfadel Cherkaoui Street, 10005, Rabat, Morocco; Cardiology A Department, Ibn Sina University Hospital Center, Mohammed V University of Rabat, Lamfadel Cherkaoui Street, 10005, Rabat, Morocco

**Keywords:** cardiology and cardiovascular systems

## Abstract

Primary cardiac tumors are extremely rare. Myxomas are the most common, typically affecting middle-aged women. They arise in the left atrium in 75% of cases. Right atrial myxomas are less common. The coexistence of coronary artery disease is rare but can be complex. We report the case of an 80-year-old male smoker with peripheral arterial disease and stable angina, who was diagnosed with a right atrial mass seven years earlier but initially declined surgery due to fear of the procedure. He was admitted to our hospital with crescendo angina. Echocardiography revealed a large right atrial mass suggestive of myxoma. Coronary angiography demonstrated severe multi-vessel atherosclerotic coronary artery disease, including mid circumflex occlusion. After successful balloon angioplasty, he underwent combined myxoma resection and coronary artery bypass grafting. This case illustrates the uncommon but serious combination of myxoma and acute coronary syndrome, reinforcing the need for careful surgical planning to achieve a favorable outcome.

## Introduction

Primary cardiac tumors are exceptionally rare, with reported incidence in the general population ranging from 0.0017% to 0.19% [1]. Among them, myxomas are the most frequent, predominantly affecting middle-aged women, with an average onset at 56 years [2]. In 75% of cases, they originate in the left atrium, typically from the interatrial septum near the fossa ovalis [1]. Right atrial myxomas (RAM) are less common and tend to occur more frequently in males [3]. Although the coexistence of coronary artery disease (CAD) has been reported, it remains infrequent. We report a challenging case of right atrial myxoma associated with acute coronary syndrome (ACS).

## Case report

We report the case of an 80-year-old male smoker with a history of peripheral arterial disease. He had been experiencing Canadian Cardiovascular Society (CCS) class II angina for seven years. At that time, transthoracic echocardiography revealed a 22 × 28 mm mass in the right atrium. Coronary angiography and cardiac surgery were recommended; however, the patient declined these interventions due to procedural apprehension and was subsequently lost to follow-up. He currently presents with crescendo angina.

Upon admission, the patient presented with a blood pressure of 130/70 mmHg, a regular pulse of 70 beats per minute, a respiratory rate of 20 breaths per minute, and an oxygen saturation of 96% on room air. Cardiac auscultation revealed normal first and second heart sounds, with no audible murmurs, rubs, or gallops. The femoral pulses were absent.

**Figure 1:**
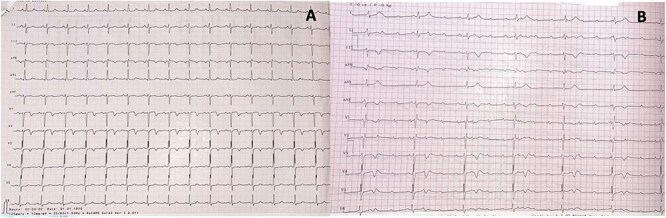
(A) Electrocardiogram on admission showing a regular sinus rhythm with a ventricular rate of 75 beats per minute, a normal axis, a PR interval of 180 ms, and T wave inversion in the anteroseptal leads. (B) Complete atrioventricular block.

Electrocardiogram (EKG) showed T wave inversion in the anteroseptal leads (Fig. 1A).

**Figure 2:**
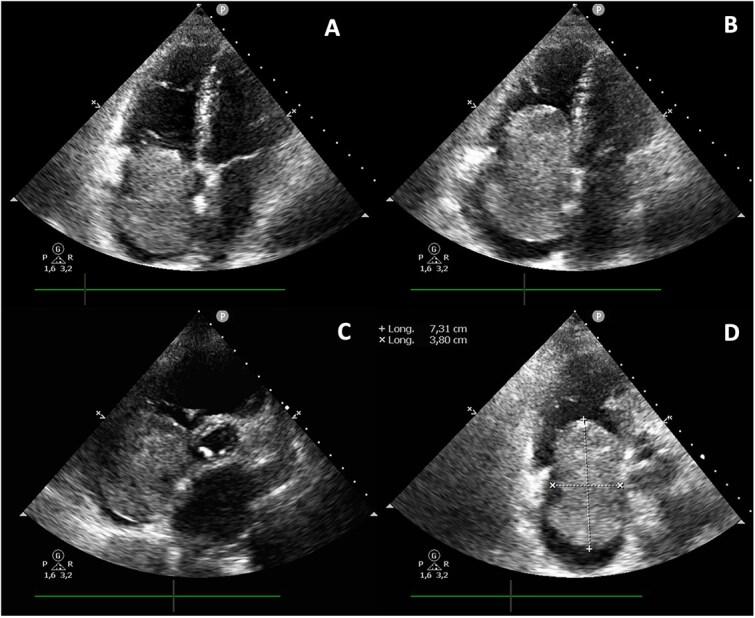
Echocardiographic evaluation revealing a large (73 × 38 mm), pedunculated, and hyperechogenic mass attached to the interatrial septum, occupying most of the right atrium and extending toward the tricuspid valve, visualized in the transthoracic apical 4-chamber view (A, B), parasternal short-axis view (C), and a modified apical 4-chamber view focused on the right chambers (D).

Troponin I level was elevated at 8 ng/mL (normal ≤ 0.04 ng/mL), confirming the diagnosis of NSTEMI.

Transthoracic echocardiography revealed a large (73 × 38 mm), pedunculated and hyperechogenic mass attached to the interatrial septum, occupying most of the right atrium and moving toward the tricuspid valve (Fig. 2), highly suggestive of a right atrial myxoma. The left ventricular ejection fraction was 52% with hypokinesis of basal and mid segments of the inferolateral, inferoseptal, and inferior walls.

**Figure 3:**
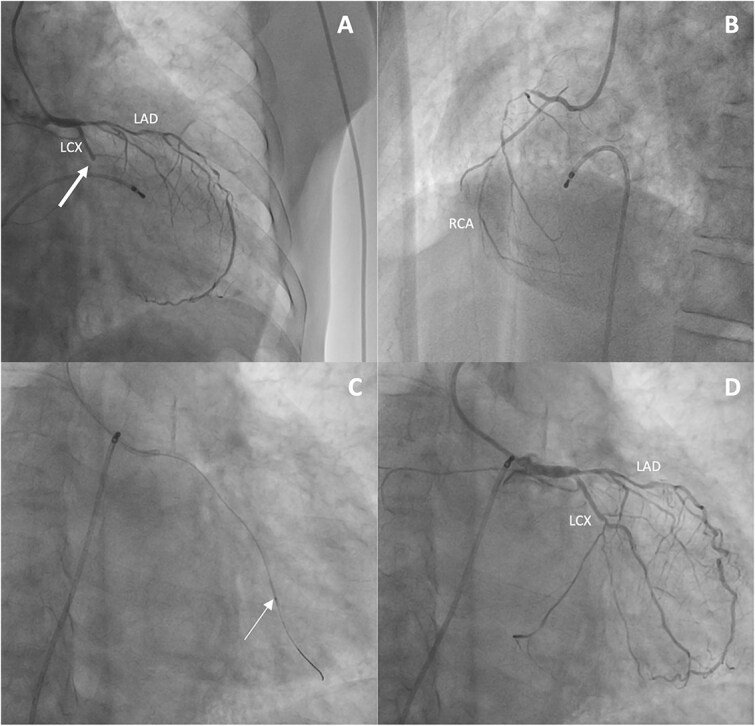
A: Coronary angiogram in the right anterior oblique view showing complete occlusion of the LCX (white arrow) and multiple critical stenoses in the LAD with preserved TIMI III flow. B: Coronary angiogram in the left anterior oblique view demonstrating a non-dominant, diffusely diseased RCA. C: Balloon (white arrow) angioplasty of the circumflex artery. D: Restoration of TIMI III flow. LAD: Left anterior descending artery; LCX: Left circumflex artery; RCA: Right coronary artery.

Shortly after echocardiography, the patient developed recurrent angina with diaphoresis. EKG showed complete atrioventricular block (Fig. 1B). He was promptly transferred to the catheterization laboratory, where monitoring revealed new ST segment elevation in inferior leads. A temporary pacing lead was difficultly implanted. Coronary angiogram indicated severe, diffuse multi-vessel atherosclerotic CAD, with complete occlusion of the mid circumflex artery (LCX) (Fig. 3A). The left anterior descending artery (LAD) showed multiple critical stenoses along its entire length, but with preserved TIMI III flow (Fig. 3A). The right coronary artery (RCA) was non-dominant and diffusely affected (Fig. 3B). Successful balloon angioplasty of the circumflex artery was performed, restoring TIMI III flow (Fig. 3C and D), with subsequent clinical improvement and return to baseline sinus rhythm.

**Figure 4:**
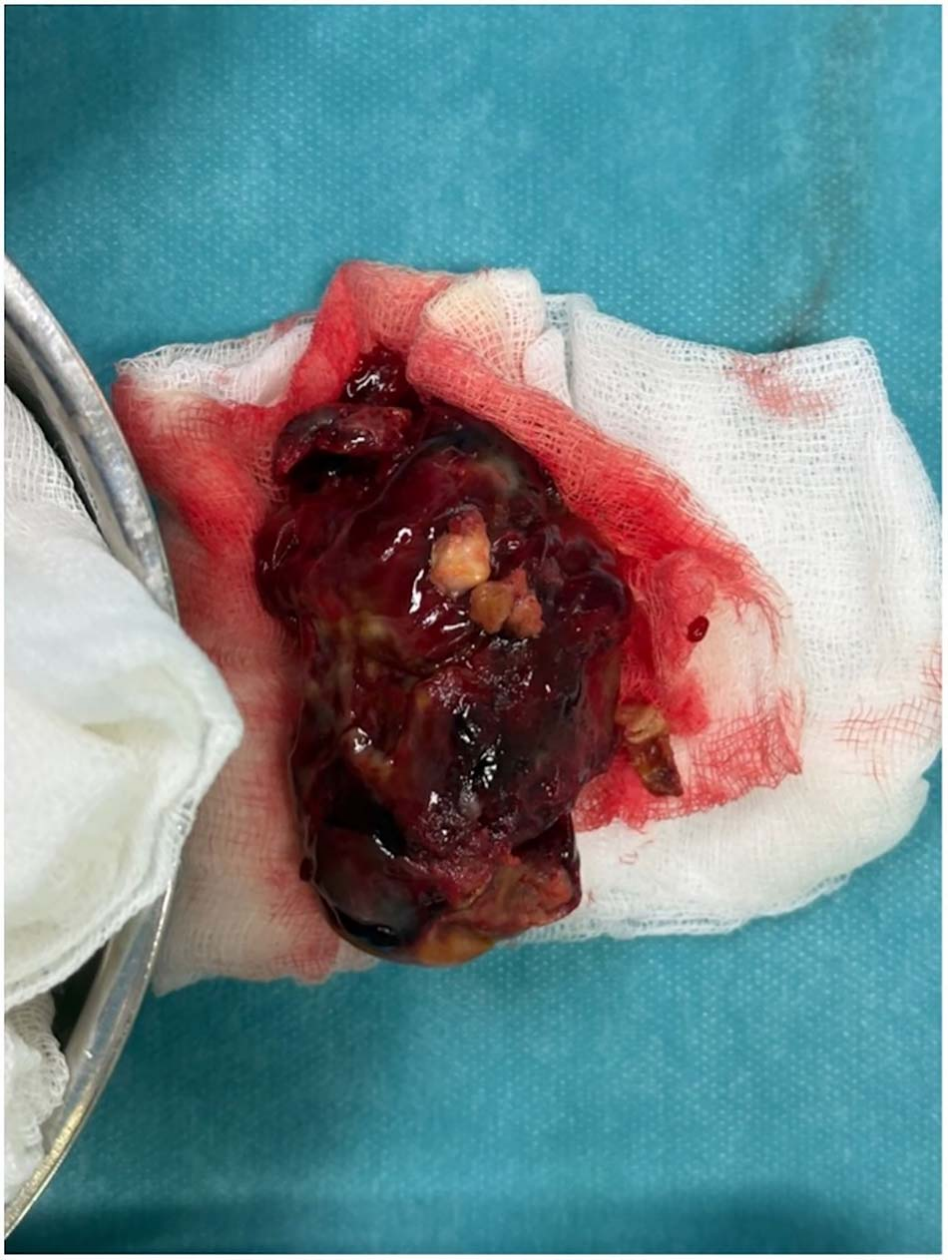
Gross specimen of the resected tumor, measuring almost 8 × 4 cm.

The patient underwent myxoma resection and coronary artery bypass grafting, including a left internal mammary artery graft to the left anterior descending artery and a saphenous vein graft to the first marginal artery (Fig. 4). Biopsy confirmed a right atrial myxoma.

## Discussion

Right atrial myxomas (RAM) are generally asymptomatic. Our patient remained asymptomatic for seven years, with an average growth rate of 0.5 × 0.2 mm per month, which aligns with the limited data available on this matter in the literature [3]. However, similar to left atrial myxomas, they can also present with the classic Goodwin’s triad. Obstructive symptoms, including dyspnea, syncope, and sudden death, occur in 60–70% of cases. Constitutional symptoms, such as malaise, fever, and weight loss, affect 10–45% of patients. Embolic events are seen in 35% of left-sided and 10% of right-sided myxomas [1, 3].

Coronary embolization should always be considered in the setting of ACS and left heart myxoma [4], especially in the presence of embolic risk factors such as hypertension, New York Heart Association (NYHA) class II, atypical tumor location, irregular tumor surface, narrow tumor base, and elevated fibrinogen levels [5]. Although rarer, RAM can also lead to coronary embolization through a right-to-left shunt, often a patent foramen ovale [6]. There are no data in the literature regarding the risk of embolization with the temporary pacing lead. In our case, this step was particularly delicate but essential.

Our patient had cardiovascular risk factors, a history of peripheral arterial disease, angina, and diffuse atherosclerotic lesions on coronary angiography strongly suggesting an atheromatous origin rather than myxoma embolization.

Coronary artery disease (CAD) prevalence in myxoma patients remains uncertain, with studies indicating a wide variability between 10.5% and 82% [7–9]. Silva et al., in a systematic review and meta-analysis of 109 patients with cardiac myxoma, reported a CAD prevalence ranging from 5.3% to 36.3%, with an average of 20.7% [2].

Although not fully understood, the association between these conditions may be mainly due to the fact that they tend to occur at similar ages [10]. Additionally, some authors suggest that patients with myxoma may exhibit elevated levels of interleukin-6 and interleukin-8 leading to a hypercoagulable state [9].

The coexistence of these conditions raises important considerations, particularly regarding the indication for preoperative coronary angiography and its impact on management. Beyond acute cases like ours, there is no clear consensus. Some authors advocate routine angiography, while others recommend it only for patients over 35–40 years old, with a history of atherosclerotic risk factors, angina, or prior myocardial infarction [2]. We believe that a computed tomographic coronary angiogram may be a good alternative in the absence of warning signs.

Patients with stable CAD can undergo simultaneous coronary bypass and tumor resection; however, no clear guidelines exist for acute cases. In our case, given the widespread coronary damage and significant improvement after balloon angioplasty, we found it reasonable to refrain from stenting and opt for combined surgery.

## References

[1] Lazaros G, Latsios G, Tsalamandris S, Sfyras N, Toutouzas K, Tsiamis E, et al. Cardiac myxoma and concomitant myocardial infarction: embolism, atherosclerosis or combination? Int J Cardiol 2016;205:124–6. 10.1016/j.ijcard.2015.11.057 26730843

[2] Silva M, Carneiro M, Nunes J, da Silva A, de Sousa M. Systematic review and meta-analysis of prevalence of coronary artery disease in adult patients with cardiac myxomas. F1000Res 2015;4:194. 10.12688/f1000research.6641.1 28620449 PMC5461895

[3] Gewehr DM, Neiverth A, Cavalcanti MS, Maestri TC, Haurani S, Kubrusly FB, et al. Fast growth rate of a right atrial myxoma. Einstein (Sao Paulo) 2022;20:eRC6478. 10.31744/einstein_journal/2022RC6478 35352769 PMC8932729

[4] Kocaturk H, Karaman A, Bayram E, Çolak MC, Yurtman V. Left atrial myxoma and concomitant atherosclerotic coronary artery disease. Eur J Med 2009;41:202–4. PMC4261271 25610104

[5] Liu Y, Wang J, Guo L, Ping L. Risk factors of embolism for the cardiac myxoma patients: a systematic review and meta-analysis. BMC Cardiovasc Disord 2020;20:348. 10.1186/s12872-020-01631-w 32711463 PMC7382866

[6] Vondran M, Ghazy T, Andrási TB, Rastan AJ. ST-segment elevation myocardial infarction and right atrial myxoma. Thorac Cardiovasc Surg Rep 2022;11:e33–7. 10.1055/s-0042-1749211 35795298 PMC9252612

[7] Fueredi GA, Knechtges TE, Czarnecki DJ. Coronary angiography in atrial myxoma: findings in nine cases. AJR Am J Roentgenol 1989;152:737–8. 10.2214/ajr.152.4.737 2784254

[8] Velasco CE, Suarez NP, Roullard CP, McCullough PA, Roberts WC, et al. Usefulness of coronary angiography in patients with left atrial myxoma. Proc (Bayl Univ Med Cent) 2020;33:529–31. 10.1080/08998280.2020.1776024 33100521 PMC7549987

[9] Li AH, Liau CS, Wu CC, Chien KL, Ho YL, Huang CH, et al. Role of coronary angiography in myxoma patients: a 14-year experience in one medical center. CCardiology 1999;92:232–5. 10.1159/000006979 10844382

[10] Garg A, Agrawal D, Sharma GL. Right atrial myxoma with coexistent coronary artery disease - a rare combination. J Cardiovasc Echogr 2020;30:100–3. 10.4103/jcecho.jcecho_73_19 33282648 PMC7706367

